# The spread of highly pathogenic avian influenza virus is a social network problem

**DOI:** 10.1371/journal.ppat.1013233

**Published:** 2025-07-07

**Authors:** Jamie Dunning, Josh A. Firth, Alastair I. Ward

**Affiliations:** 1 School of Biology, University of Leeds, Leeds, United Kingdom; 2 Department of Biology, University of Oxford, Oxford, United Kingdom; University of California San Diego, UNITED STATES OF AMERICA

## Abstract

Despite identification of Highly Pathogenic Avian Influenza viruses nearly 75 years ago, the transmission pathways among wild animals remain incompletely described. We propose the use of social networks, to complement phylodynamic modeling, for better surveillance, prediction, and prioritization of HPAI.

New types of Highly Pathogenic Avian Influenza (HPAI) have emerged repeatedly and unpredictably in recent years, and in greater numbers of novel host species [1–3], each with differing spatial and social ecologies. The spread of HPAI through wild animal communities cannot thus be explained by viral fitness alone, and must also be shaped by the composition and structure of host social networks and their physical environments [4]. However, the role of these network structures in HPAI spread remains poorly described. The ongoing panzootic of the HPAI A(H5N1) subtype and the associated risk of zoonotic transfer [2], has underscored the cost of further neglecting this knowledge gap; prompting, in June 2024, the addition of influenza A viruses to the World Health Organization’s list of pathogens of pandemic risk, and a call for further research into the role of bird migration in their spread4.

Following its detection in poultry in 1959 [2], HPAI A(H5N1) gradually became established in wild ducks, geese, and swans (Anseriformes) across Eurasia with little spill-over into other groups of birds [2,5]. Then, around 2020, changes in the HPAI A(H5N1) genome broadened the list of susceptible host taxa to include colonial seabirds (Charadriiformes) [2,6,7], but also other groups, with 356 bird species known to be affected by 2023 [1]. Spread beyond Eurasia, to the Americas, was concurrent with sustained disease prevalence outside of the non-breeding season and spread beyond birds, with infection and mortality in domestic and wild mammals [8–10]. Recent spread between cattle in the USA has for the first time provided clear evidence of mammal-to-mammal transmission of a HPAI A(H5N1) sub-type [9–11] (including via unpasteurized milk [2,9]). This raises the prospect of further mutation and reassortment within a mammalian host, and further spread amongst mammals.

Viral spread is typically monitored using molecular approaches that rely on continuous sampling of species associated with outbreak events to measure phylogenetic relationships between virus variants. Theoretically, these relationships can then be compared to reconstruct their evolutionary history and past transmission pathways. However, the large number of interacting host species, with varied behavioral ecologies, population sizes, disease prevalence and immunocompetencies within bird communities makes comprehensive sampling extremely challenging, constraining viral phylogenetic modeling. For example, large genetic distances between viral samples may involve long chains of transmission between species, where intermediate species are not sampled [12]. This gap could be filled using, global citizen science bird occurrence repositories, such as eBird [13], that can provide rich data on the structure of bird social networks at multiple spatial scales. We suggest that HPAI spread can be intuitively considered from a social network perspective—where edges represent an appropriate mode of disease transmission. Alongside viral genetic distance data and the temporal and geometric spread of sampling effort, social networks could help identify species involved in complex chains of transmission but not sampled in viral phylogenetic trees.

Social network theory provides a standardized framework for quantifying relationships between actors within a network, which is defined by a set of nodes and interconnecting edges. In this context, the nodes represent individuals or species. Edges in social networks designed to model potential disease transmission, must represent a mechanism of transmission between actors, for example, physical interaction (contact), co-occurrence as a proxy for physical contact, or shared habitat use (for passive vectors—standing water or fomites). Nodes and edges can also be attributed with other information to inform transmission risk scenarios, for instance, behavioral or spatial predispositions to HPAI exposure (association with wetlands, or scavenging), or genetic distances between virus subtypes associated with specific species. The structure of a network can be used to estimate disease spread under these different risk scenarios. For example, each node’s position within the network describes how connected or central they are to all other nodes; a measure of their importance to network structure. In social networks designed to measure pathogen transmission, node-level centrality metrics are used to estimate specific contributions to outbreak events. For example, nodes with high ‘betweenness’ centrality (i.e., those that bridge network structure and provide pathways between otherwise unconnected nodes), may be more likely to be influential in the spread of HPAI widely and across species which are not directly in social contact with one another.

The use of social networks for the surveillance of infectious disease is well-established, particularly in human systems, and recent advances allow predictive models to incorporate direct and indirect modes of transmission. For example, during the 2019–2023 COVID-19 pandemic, real-world human social association (those without physical interaction) networks were used to examine the efficacy of contact tracing and case isolation to mitigate disease transmission [14]. In systems of wild animals, networks with edges representing social associations between individuals or species have been similarly applied [15]. While social networks in isolation can only suggest potential routes of transmission, their ability to predict patterns of HPAI spread could be improved by integrating with viral phylogenetic modeling, enabling comparison between potential transmission pathways and real-world outbreak events (and across more species than current viral phylogenetic data would allow). For example, recent predictions of zoonotic disease in mammals were derived by combining species range overlap networks with viral genetic distances to identify virus ‘reservoirs’ (from species’ centrality within these networks) and to highlight possible transmission routes through related taxa [15]. Further, and in relation to HPAI, previous studies have used shared flyway information to suggest links between migratory phenology and HPAI outbreaks in poultry [16,17]. Nevertheless, while these previous studies have been primarily limited to leveraging shared flyway information or GPS-tracking data to link migratory phenology with HPAI outbreaks in poultry, future work could now build upon these foundations by integrating viral phylogenetics with comprehensive social network analyses applied to broad-scale co-occurrence data, either through network attribution, or by modeling centrality against viral genetic distance (where sampling allows). Such studies could be used to suggest transmission routes at both landscape and community scales.

The application of social networks to the complex problem of global HPAI spread among wild birds (and mammals) presents a promising opportunity to prioritize transmission pathways for intervention, by evaluating and weighting all possible routes of transmission based on proxies of social association between species and individuals. To achieve this, we specifically propose the use of nested social networks (Fig 1) at three spatial scales: (1) within local communities (for example, within a farm or wetland) where edges represent direct interactions between individuals (for example, data on physical contact or co-feeding); (2) between species at landscape scales, where edges represent larger-scale social associations between species, such as co-occurrence in the same place at the same time (for example, inferred from surveys, bird ringing/banding records, etc.); and (3) between species that share flyways at intercontinental scales, where edges represent the shared migratory routes that link regional and local transmission networks (for example, records spread along flyways).

**Fig 1:**
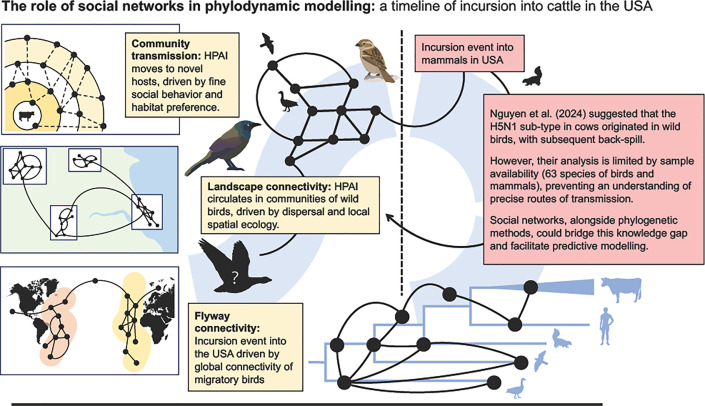
Left: A series of simplified animal social networks, at the international, landscape, and community scales, to illustrate how social structure, at each scale may contribute to disease spread. Using networks in this way captures almost all possible routes of transmission between species and can be used in predictive modeling, especially when combined with complementary viral phylogenetic modeling and as part of broad phylodynamic study. Right: A simplified evolutionary history of HPAI A(H5N1) 2.3.4.4b B3.13 (‘cow flu’) as proposed by Nguyen and colleagues [11]—showing that the viral genome that emerged in mammals in the USA was likely derived from wild birds. A superimposed social network illustrates how social network may work alongside reactive viral phylogenetics.

We propose that social networks of wild birds and analysis of network position metrics as proxies for community structure can overcome some of the current limitations by using data across spatial and temporal scales. While data for many taxonomic groups are not suitable for multi-scale modeling of social networks, there is an abundance of suitable presence data available for wild birds [12,13] at all but the local community scale. At very fine scales (for example, on specific farms) focused surveys are still required to measure physical contact or close proximity. In particular, ‘complete lists’ of all species recorded at given locations at given times, represent a simple measure of community structure to which social network analysis can be applied.

Datasets for the UK include the British Trust for Ornithology’s Wetland Bird Survey (WeBS) and BirdTrack, and internationally eBird, maintained by the Cornell Lab of Ornithology. The latter represents the largest repository of complete list data, with 600 million bird observations submitted globally, albeit with greater densities of recording across Europe, North America, and Australasia. eBird, has already been used to link historical relative abundance of wild birds with concurrent HPAI spread to poultry [12]. These, and similar, data are also appropriate for reconstructing co-occurrence networks for past HPAI outbreaks, with the resultant network structure then forming the basis of predictive transmission models [16,17]. Spatial and temporal scales over which these models can reasonably be expected to predict require evaluation. Nevertheless, the high frequency and abundance of bird record submission for many countries to repositories such as eBird offers potential to build new social network models rapidly for comparison with emerging virus phylogenetic data, or even informing on where (and which species) further viral sampling efforts may be best suited.

During 2024, phylogenetic models were used to map past transmission of HPAI A(H5N1) between wild birds and cows in the USA, and then, from cows back into communities of wild birds [11] (Fig 1). The routes of transmission included several important gaps where intermediate species were likely involved in transmission chains that, we propose, could be identified by multi-scale wild bird social network models. We argue that combining social networks across the scales presented here, in tandem with viral phylogenetic modeling, could generate new insight into the transmission of HPAI through complex networks of wild animals.
